# Morphological and bone strength indices in girls with adolescent idiopathic scoliosis and their correlations with leptin and soluble leptin receptor

**DOI:** 10.1186/1748-7161-10-S1-O13

**Published:** 2015-01-19

**Authors:** Elisa MS lam, Wayne Lee, Ka-yee Cheuk, Tsz-Ping Lam, Bobby KW Ng, Simon KM Lee, Yong Qiu, Jack CY Cheng

**Affiliations:** 1 Department of Orthopaedics and Traumatology, The Chinese University of Hong Kong, Hong Kong; 2 Spine Surgery, The Affiliated Drum Tower Hospital of Nanjing University Medical School, Nanjing, China; 3 Lee Hysan Clinical Research Laboratories, The Chinese University of Hong Kong, Hong Kong; 4 Joint Scoliosis Research Center of the Chinese University of Hong Kong and Nanjing University China

## Objective

Previous studies suggested that leptin has profound effects on bone metabolism and growth. Abnormal leptin and soluble leptin receptor (sOB-R) levels and their correlation patterns with bone mineral density and trabecular bone micro-architecture were recently found to be distinct in girls with adolescent idiopathic scoliosis (AIS). Structural Model Index (SMI) and data derived from Finite Element Analysis (FEA) are important HR-pQCT parameters that can provide important information on the rod/plate-like configurations in the trabecular bone and bone strength respectively. This study aimed to compare the differences and correlations between SMI, bone strength indices and leptin and sOB-R between AIS and controls.

## Material and methods

104 AIS girls aged 12 to 14 and 82 age and gender-matched healthy controls were recruited. Subjects with BMI>23.0 kg/m2 were excluded. Anthropometric measurements including body height, body weight, sitting height and arm span were recorded. Sexual maturation was assessed with Tanner stages. SMI and bone strength parameters from FEA were determined at the non-dominant distal radius using HR-pQCT Serum total leptin and sOB-R levels were measured with ELISA.

## Results

Compared with controls, AIS subjects had higher sOB-R level (p=0.006), higher SMI value (p=0.020) reflecting more rod-like structures within the trabecular compartment, and numerically lower stiffness (-2.03%) and estimated failure load (-3.07%). Significant negative correlation was found between SMI and serum total leptin level in AIS (r=-0.325; p=0.003) but not in controls (p=0.533). Significant positive correlations were found between stiffness, estimated failure load, and serum total leptin in both AIS (r=0.278, p=0.003; r=0.268, p=0.004 respectively) and controls (r=0.462, p<0.001; r=0.468, p<0.001 respectively).

## Conclusion

The higher SMI and numerically lower FEA derived bone strength parameters both reflecting decreased bone strength in AIS. The negative correlation between SMI and serum total leptin level was distinctly only detected in AIS, which indicated possible disturbance in leptin signaling affecting the trabecular bone of AIS. The results of this and previous studies provided strong evidences of deranged bone quality and bone strength and its association with abnormal leptin bioavailability and signaling in AIS.

